# Draft genome of the *Leptospira interrogans* strains, Acegua, RCA, Prea, and Capivara, obtained from wildlife maintenance hosts and infected domestic animals

**DOI:** 10.1590/0074-02760160010

**Published:** 2016-04

**Authors:** Frederico S Kremer, Marcus R Eslabão, Sérgio Jorge, Natasha R Oliveira, Julia Labonde, Monize NP Santos, Leonardo G Monte, André A Grassmann, Carlos EP Cunha, Karine M Forster, Luísa Z Moreno, Andrea M Moreno, Vinicius F Campos, Alan JA McBride, Luciano S Pinto, Odir A Dellagostin

**Affiliations:** 1 Universidade Federal de Pelotas, Centro de Desenvolvimento Tecnológico, Núcleo de Biotecnologia, Pelotas, RS, Brasil; 2 Universidade de São Paulo, São Paulo, SP, Brasil

**Keywords:** Leptospirosis, genomics, neglected diseases, bioinformatics

## Abstract

In the present paper, we announce new draft genomes of four *Leptospira interrogans* strains named Acegua, RCA, Prea, and Capivara. These strains were isolated in the state of Rio Grande do Sul, Brazil, from cattle, dog, Brazilian guinea pig, and capybara, respectively.

The *Leptospira* genus comprises at least 22 different species, some of which, like *Leptospira interrogans*, *Leptospira borgpetersenii*, *Leptospira santarosai*, *Leptospira noguchii*, and *Leptospira kirschneri*, are pathogenic and may cause leptospirosis (Boonsilp et al. 2013,Bourhy et al 2014). This neglected zoonosis is globally distributed and has become a reemerging public health problem in many countries, with stronger impact in tropical regions (Evangelista & Coburn 2010, Guerra 2013). Commonly found in rodents, leptospires may also infect and be hosted by different domestic and wildlife animals (Bharti et al. 2003). This wide variety of reservoirs may play a key role in the maintenance and transmission of the disease (Levett 2001). Therefore, genome sequencing of isolates from different hosts potentially provides a starting point to towards understanding the ability of *Leptospira* spp to adapt to specific host and the basis of pathogen-host interaction.

In the present study, whole-genome sequencing was performed for the strains Acegua, isolated from a stillborn bovine foetus (Monte et al. 2015), RCA, isolated from a domestic dog with clinical leptospirosis, Prea, isolated from Brazilian guinea pig (*Cavia aperea*) (Monte et al. 2013), and Capivara, isolated from capybara (*Hydrochoerus hydrochaeris*) (Jorge et al. 2012).

The isolates were cultured in Ellinghausen-McCullough-Johnson-Harris (EMJH) medium supplemented with 10% *Leptospira* enrichment EMJH (Difco, USA), 200 μg/mL 5-fluorouracil, and 5% foetal calf serum in an incubator at 30ºC without agitation. DNA extraction was performed using the commercial Illustra Bacteria GenomicPrep Mini Spin kit (GE Healthcare, USA), following the manufacturer instructions.

The whole genome sequences were obtained using an Illumina MiSeq paired-end library for Acegua, an Illumina MiSeq paired-end library and an Ion Torrent PGM fragment library for RCA and Prea, and an Ion Torrent PGM fragment library for Capivara. The raw reads were filtered by quality using Fastx-Toolkit (hannonlab.cshl.edu/fastx_toolkit/) and the paired-end reads were trimmed using Trimmomatic (Bolger et al. 2014).

*De novo* assembly was performed using A5 (Tritt et al. 2012), SGA (Simpson & Durbin 2012), and Ray (Boisvert et al. 2010) for Acegua, A5, SGA, Ray, MIRA (chevreux.org/), Newbler (roche.com/), and SPAdes (Bankevich et al. 2012) for RCA and Prea, and MIRA, Newbler, and SPAdes for Capivara. For each strain the *de novo* assemblies were merged using CISA (Lin & Liao 2013) and evaluated using QUAST (Gurevich et al. 2013). Genome annotation was performed as previous described (Kremer et al. 2015) using Prodigal (Hyatt et al. 2010), NCBI-BLAST+ (Altschul et al. 1990, Camacho et al. 2009), Uniprot (Apweiler et al. 2004), HMMER (Eddy 2011), AntiFam (Eberhardt et al. 2012), tRNAscan-SE (Lowe & Eddy 1997), RNAmmer (Lagesen et al. 2007), INFERNAL (Nawrocki et al. 2009), Aragorn (Laslett 2004), and Rfam (Griffiths-Jones et al. 2003), and manually reviewed using Artemis (Rutherford et al. 2000).

*In silico* multilocus sequence typing (MLST) was performed using BLASTn from the NCBI-BLAST+ and allele data from the *Leptospira* MLST scheme 1 (Boonsilp et al. 2013), obtained from PubMLST repository (pubmlst.org/).

The results of the *de novo* assemblies are presented in Table I. The isolates were initially sequenced using only the Illumina platform, but the high fragmentation in the resulting assembly for Prea and RCA isolates (data not showed) motivated the use of a second next-generation sequencing technology to improve the original draft sequences. Although usually not required, the combination of data of two or more platforms in the sequencing of a given genome may result in a more accurate assembly, considering that each sequencing technology has it owns bias. The most common errors associated with Illumina data occurs on CG-poor and CG-rich regions, while IonTorrent, duo to its chemistry, has a high error-rate in homopolymeric regions. In fact, both characteristics are found in*Leptospira* genomes.

**TABLE I:** Summary of the assembly results

Isolate	Scaffolds^*a*^(n)	Assembly length (Mb)	N50 (bp)	CG (%)
Acegua	158	4.6	63,489	35.07
RCA	89	4.43	55,782	35.06
Prea	106	4.44	46,508	35.17
Capivara	160	4.51	45,526	34.98

*a*: all assembled sequences joined (or not) by linkage information.

During genome annotation (Table II), by using our pipeline, in addition to the coding DNA sequences, we were also able to identify many noncoding feature in all four genomes, including not only transfer RNAs and ribosomal RNAs, but also transfer-messenger RNAs (tmRNAs), RNase P *loci*, and riboswitches. There is an increasing interest in the analysis of gene expression in*Leptospira*, especially during infection (Matsui et al. 2012, Lehmann et al. 2013, Caimano et al. 2014, Eshghi et al. 2014). Recent studies have already performed whole-transcriptome sequencing of *L. interrogans* and many noncoding features associated with gene expression regulation and transcriptional/translational processing were identified, including RNase P, tmRNAs, riboswitches, as well other families of noncoding RNA. Therefore, the identification of noncoding features in the annotation of newly sequenced genomes may allow a more accurate description of the resulting transcriptome.

**TABLE II:** Summary of the annotation results

Isolate	CDS	tRNAs	rRNAs	Other ncRNAs^*a*^	Riboswitches
Acegua	3734	37	4	2	3
RCA	3591	34	3	10	5
Prea	3616	33	5	10	3
Capivara	4146	37	3	7	3

*a*: includes the noncoding RNAs identified by Rfam and Aragorn; CDS: coding DNA sequence; ncRNAs: noncoding RNA; rRNAs: ribosomal RNAs; tRNAs: transfer RNAs.

The *in silico* MLST sequence types (ST) for the four isolates are presented in Table III. Previously identified by variable-number tandem-repeat as*L. interrogans* serogroup Australis serovar Muenchen (Monte et al. 2015), the Acegua isolate was a match for ST24 that contains two *L. interrogans* serogroup Australis isolates, while the Capivara isolate was identified as ST17 that includes nine *L. interrogans* serogroup Icterohaemorrhagiae isolates (5 belonging to serovar Copenhageni and 2 to serovar Icterohaemorrhagiae). Preliminary analysis revealed that the *pfkBlocus* was absent in the draft assemblies of RCA and Prea. To investigate this fact, the raw reads from these isolates were aligned using BLASTn against a reference set of *pfkB* alleles obtained from the PubMLST repository. The BLAST XML output was analysed by a Python script to identify reads that correspond to this *locus* using an identity threshold of 95%. The selected reads were saved in FASTQ format and filtered by quality using a minimum Phred score of 20 in at least 95% of the bases. After filtering, 83 reads remained in the Prea set, and 90 in the RCA set, corresponding to mean coverages of about 18 and 20-fold, respectively. Therefore, the absence of this *locus* in both draft genomes was a result of an assembly artifact. For each genome, the reads that aligned to the *pfkB* database were assembled using CAP3 (Huang & Madan 1999) and the resulting contigs were aligned against the same database to identify the corresponding alleles in the MLST scheme 1, that are showed in Table III.

**TABLE III:** Sequence types (ST) profiles of the Acegua, RCA, Prea, and Capivara strains based on the *Leptospira* multilocus sequence typing scheme 1

Isolate	*glmU*	*pntA*	*sucA*	*tpiA*	*pfkB*	*mreA*	*caiB*	ST
Acegua	1	4	2	1	5	3	4	24
RCA	1	1	2	2	10^*a*^	4	8	17
Prea	1	1	2	2	10^*a*^	4	8	17
Capivara	1	1	2	2	10	4	8	17

*a*: hit not found in the BLAST against the draft genome assembly.

The *Leptospira* genus comprises more than 300 serovars and pathogenic species were already reported in a wide variety of animal hosts. However, from the 233 genome sequences indexed in BioProject database and available at GenBank with host information, the major part (166) was obtained from human samples (ncbi.nlm.nih.gov/bioproject/). The sequencing of isolates obtained from wildlife animals, like *C. aperea* and *H. hydrochaeris*, both rodents and natural reservoirs, provide data for future pangenome and pathogenome analysis intending to understand the factors that guide the pathogen-host interactions. Additionally, the isolate Acegua, obtained from a bovine stillborn, also represents an interesting source of information about these interactions, since abortion induced by leptospirosis in cattle is usually associated to the serovar Hardjo of the species*L. interrogans* and *L. borgpetersenii*, not to Muenchen, although this serovar has been associated to abortions in pigs (Ellis et al. 1986).

Finally, the analysis of these isolates also provide new insights into the serogroups circulating in the south of Brazil, suggesting that while *L. interrogans* serogroup Icterohaemorrhagiae serovars Icterohaemorrhagiae and Copenhageni are present, they are not the only ones. Based on the MLST profiles, serovars belonging to serogroup Australis are also circulating among wild and domestic animals, and the comparative analysis of genomic data may be applied to trace their distribution and evolution. Furthermore, the availability of these new genome sequences from four *L. interrogans* strains, isolated from diverse hosts, will provide useful data towards understanding the molecular diversity and pathogenesis of these new strains.

*Nucleotide sequence accessions* - These Whole Genome Shotgun projects have been deposited at DDBJ/EMBL/GenBank under the accessions LCZF00000000 for Acegua, LJBP00000000 for RCA, LJBO00000000 for Prea, and LJBQ00000000 for Capivara. The versions described in this paper are LCZF01000000, LJBP01000000, LJBO01000000, and LJBQ01000000, respectively.
